# Improvement in verbal learning over the first year of antipsychotic treatment is associated with serum HDL levels in a cohort of first episode psychosis patients

**DOI:** 10.1007/s00406-019-01017-w

**Published:** 2019-04-27

**Authors:** Priyanthi B. Gjerde, Carmen E. Simonsen, Trine V. Lagerberg, Nils E. Steen, Torill Ueland, Ole A. Andreassen, Vidar M. Steen, Ingrid Melle

**Affiliations:** 1 NORMENT, Department of Clinical Science, University of Bergen, 5021 Bergen, Norway; 2 Dr. Einar Martens Research Group for Biological Psychiatry, Department of Medical Genetics, Haukeland University Hospital, 5021 Bergen, Norway; 3 NORMENT, Oslo University Hospital, 0424 Oslo, Norway; 4 Department of Clinical Medicine, University of Oslo, 0424 Oslo, Norway; 5 Department of Psychology, University of Oslo, 0317 Oslo, Norway; 6 Laboratory Building, Department of Clinical Science, Haukeland University Hospital, P.O. Box 7804, 5021 Bergen, Norway; 7 Oslo University Hospital, Ullevaal, Building 49, Nydalen, P.O. Box 4956, 0424 Oslo, Norway

**Keywords:** Schizophrenia, Psychosis, Antipsychotics, Lipids, Cognition, Outcome

## Abstract

To investigate whether changes in serum lipids are associated with cognitive performance in first episode psychosis (FEP) patients during their first year of antipsychotic drug treatment. One hundred and thirty-two antipsychotic-treated FEP patients were included through the TOP study along with 83 age- and gender-matched healthy controls (HC). Information regarding cognitive performance, psychotic symptoms, lifestyle, body mass index, serum lipids [total cholesterol, high-density lipoprotein (HDL) cholesterol, low-density lipoprotein cholesterol, and triglycerides] and antipsychotic treatment was obtained at baseline and after 1 year. The cognitive test battery is comprised of assessments for verbal learning, processing speed, working memory, verbal fluency, and inhibition. Mixed-effects models were used to study the relationship between changes over time in serum lipids and cognitive domains, controlling for potential confounders. There was a significant group by HDL interaction effect for verbal learning (*F* = 11.12, *p* = 0.001), where an increase in HDL levels was associated with improvement in verbal learning in FEP patients but not in HC. Practice effects, lifestyle, and psychotic symptoms did not significantly affect this relationship. Antipsychotic-treated FEP patients who increased in HDL levels during the first year of follow-up exhibited better verbal learning capacity. Further investigations are needed to clarify the underlying mechanisms.

**Electronic supplementary material:**

The online version of this article (10.1007/s00406-019-01017-w) contains supplementary material, which is available to authorized users.

## Introduction

More than 70% of patients with schizophrenia are estimated to experience cognitive impairments [1]. For several cognitive domains—including verbal learning and memory, attention, perception, and processing speed—the impairment may be as prominent as two standard deviations below healthy controls [2] indicating a clinically relevant decrease or deficits in cognitive functioning. These deficits can have a more substantial impact than positive psychotic symptoms in determining real-world functional outcome [3, 4], and may be present even after successful reduction in psychotic symptoms [5, 6]. Although considerable efforts have been made to understand the neurobiology of cognitive impairment in psychosis, the background is still mostly undetermined.

Lipids are essential for CNS development and functioning [7–10], including myelination and synapse communication. In schizophrenia and related psychotic disorders, both lipid disturbances as well as brain structural changes have been demonstrated. While several studies in the normal aging population, as well as in other neuropsychiatric disorders [11–17], characterized by compromised brain functioning have indicated a link between serum cholesterol levels, in particular HDL cholesterol, and cognition, few studies have examined the potential relationship between serum lipids and cognition in patients with schizophrenia. In a cross-sectional study, Lancon et al. [18] found low HDL levels (along with hypertriglyceridemia, and abdominal obesity) to be related to impairment in verbal learning and memory. Another cross-sectional study [19] demonstrated that patients with schizophrenia and co-morbid metabolic syndrome exhibited impairments in processing speed, attention/vigilance, working memory and problem solving, compared to those without metabolic syndrome. However, when the authors examined the different components of the metabolic syndrome, they found that the HDL level was positively associated with attention/vigilance. Also, studies in antipsychotic-treated patients have reported a link between serum lipids and cognitive performance. Krakowski and colleagues found that an increase in total cholesterol level and LDL level after a 12-week period of antipsychotic drug treatment was positively associated with improvements in global cognition [20] with verbal memory being the most prominent domain. Still, there are other studies in schizophrenia that have failed to show any associations between serum lipids and cognition [21, 22], and some have even found negative associations between serum lipids and cognitive performance [23].

These discrepancies in prior studies may be due to the potential confounding effects of long-term medication, age-related neurodegeneration and disease risk-related environmental factors, as most previous studies have been focusing on chronic schizophrenia patients in a cross-sectional study design. Another important limitation is a lack of age- and gender-matched healthy individuals to control for practice effects [24] and naturally occurring variations in serum lipid levels.

## Aims of the study

The present longitudinal study of an FEP cohort was undertaken to investigate potential associations between serum lipid levels and cognitive performance in the first year of antipsychotic treatment in a group of FEP patients with limited prior drug exposure, compared to a group of age- and gender-matched healthy controls (HC). We further investigated the possible influence of lifestyle (smoking, alcohol, diet, and exercise), metabolic measures connected to serum lipids (body mass index), improvements in clinical symptomatology secondary to antipsychotic drug treatment, and other illness-related factors (antipsychotic drug treatment, hospitalization, and depressive symptoms) on the relationship between serum lipids and cognition in the patient group.

## Methods and materials

### Participants

The longitudinal FEP sample, recruited between 2003 and 2013 through the Thematically Organized Psychosis (TOP) study (Oslo, Norway), was the same as used by Gjerde et al. [25]. The inclusion criteria for the TOP study were age between 18 and 65 years, and having a diagnosis within a broad schizophrenia spectrum psychosis according to the Diagnostic and Structural Manual of Mental Disorders, fourth version (DSM-IV, American Psychiatric Association, 2000). Participants were recruited in a stable phase of their disorder. Exclusion criteria were current IQ under 70, the presence of a neurological disorders or a history of head injuries. Additionally, for the longitudinal FEP study, patients who previously had received adequate antipsychotic drug treatment were not recruited [25]. Healthy controls (HC) were randomly selected and recruited from the same geographic catchment area as the patients, using national statistical records (2003–2011). They were matched for age and gender. To ensure a reliable HC sample, subjects were excluded if they or any of their first-degree relatives had a history of a severe psychiatric disorder as indicated by the Primary Care Evaluation of Mental Disorders [26]. The final sample consisted of 132 first episode psychosis (FEP) patients and 83 HC. Participants were tested at baseline and after 1 year. None of the participants used medication against thyroid problems, diabetes, hypertension or cholesterol-lowering medications (statins or fibrates).

All participants gave written informed consent prior to their inclusion in the study, and the Regional Ethics Committee and The Norwegian Data Inspectorate approved the study.

### Clinical assessments

Demographic and clinical data (including antipsychotic drug treatment) were obtained through structured interviews and from hospital medical records. The diagnosis was established by use of the Structural Clinical Interview for DSM-IV (SCID) [27]. Psychotic symptoms were assessed with the Positive and Negative Syndrome Scale (PANSS) [28] and depressive symptoms with the Calgary Depression Scale for Schizophrenia (CDSS) [29]. Remission was defined as at least 1 week with no score of ≥ 4 on any of the following five PANSS items: P1, P3, P5, P6, or G9. Alcohol use was measured with the Alcohol Use Disorders Identification Test (AUDIT) [30]. Information on diet and exercise habits for the FEP patients was obtained by self-assessment at baseline and follow-up. Patients were asked to describe their eating habits as healthy, moderately healthy or unhealthy, while exercise was initially assessed as light, moderate or hard. Diet was then dichotomized into “similar or poorer diet” (including patients who did not change their diet habits from inclusion to 12 months, as well as patients who reported a poorer diet at 12 months) and “better diet” (consisting of patients who reported a beneficial change in diet). Exercise was likewise dichotomized into “similar or reduced exercise” and “increased exercise” at 12 months (for more details, see [25]).

### Antipsychotic drug treatment in the FEP group

As we did not have sufficient statistical power to create separate subgroups for the different antipsychotic agents (see supplementary Tables 1, 2 and 3 for antipsychotic use), we organized data on antipsychotic medication as “continuous antipsychotic drug use” versus “intermittent antipsychotic drug use” [25]. Continuous use was defined as using any antipsychotic agent at baseline and at 12 months (not necessarily the same agent at both time points), while intermittent use was defined as using any antipsychotic agent at either baseline or at 12 months. In addition, we differentiated between antipsychotic monotherapy (using only one antipsychotic at the specific time point) and polypharmacy (using two or more antipsychotic agents at the same time point). All patients who used first-generation drugs also used second-generation drugs, and due to insufficient information regarding switches and duration of antipsychotic treatments during the follow-up period, it was not possible to calculate cumulative drug exposure.

**Table 1:** Demographic, metabolic, and cognitive characteristics of the FEP and HC sample

	FEP (*n*)	HC (*n*)	FEP, mean (SD)	HC, mean (SD)	*t*	*p* value
Age (years)	132	83	26.7 (7.6)	29.0 (6.8)	− 2.6	**0.01**
Education (years)	132	83	12.9 (2.8)	14.2 (2.0)	− 4.43	**< 0.001**
Alcohol use (AUDIT scores)	120	71	7.3 (7.4)	5.9 (3.0)	1.89	0.06
DUP (weeks), median (range)	132		40.0 (1–1040)			
Total PANSS scores	132		63.2 (13.9)			
PANSS-positive subscores	132		2.6 (1.0)			
PANSS-negative subscores	132		2.2 (1.0)			
Depressive symptoms (CDSS scores)	128		6.2 (4.5)			

	FEP (*n*)	HC (*n*)	FEP, *n* (%)	HC, *n* (%)	*X*^2^	*p* value
Gender (male)	132	83	85 (64.4)	80 (59.7)	0.62	0.45
Ethnicity (Caucasian)	132	83	87 (65.9)	132 (98.3)	52.32	**< 0.001**
Smoking (daily)	132	86	60 (45.5)	17 (19.8)	15.04	**< 0.001**
Hospitalization at inclusion	132		30 (22.7)			
Change in diet (“better diet”)^a^	132		20 (15.2)			
Change in exercise (“increased exercise “)^b^	129		21 (15.9)			
Continuous antipsychotic drug use^c^	132		100 (76)			
Intermittent antipsychotic drug use^d^	132		32 (24)			
Antipsychotic monotherapy	132		91 (69)			
Antipsychotic polypharmacy	132		41 (31)			

*p* values are obtained from *T* tests and chi-square test

*p* values in bold indicate numbers that are significant on the 95% confidence limit

*FEP* first episode psychosis, *HC* healthy controls, *SD* standard deviation, *n* number of subjects, % percentage, *AUDIT* Alcohol Use Disorder Identification Test, *DUP* duration of untreated psychosis, *PANSS* Positive and Negative Syndrome Scale, *CDSS* Calgary Depression Scale for Schizophrenia

^a^Diet was divided into “similar or poorer” and “better” compared to diet habits at baseline

^b^Exercise was divided into “similar or reduced” and “increased” compared to exercise habits at baseline

^c^“Continuous antipsychotic drug use” refers to the continuous use of any antipsychotic agent during the study period

^d^“Intermittent antipsychotic drug use” refers to the use of any antipsychotic agent, either at baseline or 12 months

**Table 2:** Mixed models examining serum lipids, BMI and cognitive performance at baseline in FEP and HC

	FEP, mean (SD)	HC, mean (SD)	Group		Group	
			*F*	*p* value	*B*	SE (*B*)
Total cholesterol (mmol/L)	4.97 (1.00)	4.59 (0.90)	8.94	**0.003**	0.39	0.13
HDL (mmol/L)	1.39 (0.40)	1.44 (0.43)	0.08	0.79		
LDL (mmol/L)	2.99 (0.82)	2.59 (0.81)	11.54	**0.001**	0.38	0.11
Ln TG (mmol/L)	0.81 (0.31)	0.77 (0.26)	0.82	0.37		
BMI (kg/m^2^)	24.9 (4.3)	24.1 (3.7)	1.57	0.21		
Verbal learning (total scores)	49.31 (11.80)	59.22 (8.54)	35.25	**< 0.001**	− 8.82	1.48
Processing speed (total scores)	60.40 (16.82)	79.55 (13.32)	65.19	**< 0.001**	− 16.77	2.08
Working memory (total scores)	9.21 (2.61)	11.89 (2.40)	48.33	**< 0.001**	− 2.50	0.36
Verbal fluency (total scores)	35.58 (14.54)	45.39 (9.98)	18.27	**< 0.001**	− 7.65	1.79
Inhibition (total scores)	66.07 (24.14)	49.52 (10.83)	27.75	**< 0.001**	15.08	2.86

*p* values in bold indicate numbers that are significant on the 95% confidence limit

Verbal learning was measured with California Verbal Learning Test, processing speed with Digit Symbol Test, working memory with Letter number span, verbal fluency with Verbal Fluency Test, and inhibition with Color-Word Interference Test. Analyzed with mixed-effects models while controlling for age, gender, education, and group (FEP vs. HC). The HC were defined as the reference group

*FEP* first episode psychosis, *HC* healthy controls, *SD* standard deviation, *F F* value, *B* regression coefficient, *SE* standard error of the regression coefficient, *HDL* high-density lipoprotein cholesterol, *LDL* low-density lipoprotein cholesterol, *Ln TG* Ln of triglycerides, *BMI* body mass index

**Table 3:** Mixed models examining serum lipid, BMI and cognitive trajectories over 1 year in FEP and HC

	Time		Group × time		Group × time	
	*F*	*p* value	*F*	*p* value	*B*	SE (*B*)
Total cholesterol (mmol/L)	0.005	0.94	0.003	0.96		
HDL (mmol/L)	2.77	0.10	2.24	0.14		
LDL (mmol/L)	0.32	0.57	1.48	0.23		
Ln TG (mmol/L)	0.05	8.39	0.05	0.82		
BMI (kg/m^2^)	23.84	**< 0.001**	6.99	**0.01**	0.95	0.36
Verbal learning (total scores)	17.60	**< 0.001**	6.86	**0.01**	3.54	1.35
Processing speed (total scores)	0.05	**0.003**	2.27	0.13		
Working memory (total scores)	0.10	0.75	0.10	0.75		
Verbal fluency (total scores)	0.21	0.65	6.38	**0.01**	− 3.15	1.25
Inhibition (total scores)	0.60	**0.02**	0.37	0.54		

*p* values in bold indicate numbers that are significant on the 95% confidence limit

Verbal learning was measured with California Verbal Learning Test, processing speed with Digit Symbol Test, working memory with Letter number span, verbal fluency with Verbal Fluency Test, and inhibition with Color-Word Interference Test. Analyzed with mixed-effects models examining a time × group interaction while controlling for age, gender, education, time, and group (FEP vs. HC). The HC were defined as the reference group

*FEP* first episode psychosis, *HC* healthy controls, *F f* value, *B* regression coefficient, *SE* standard error of the regression coefficient, *HDL* high-density lipoprotein cholesterol, *LDL* low-density lipoprotein cholesterol, *Ln TG* ln of triglycerides, *BMI* body mass index

### Cognitive tests

The cognitive test battery is comprised of five cognitive domains (verbal learning, processing speed, working memory, verbal fluency, and inhibition) based on previous findings related to cognitive dysfunction in FEP patients [31].

Psychologists trained in standardized neuropsychological testing carried out the cognitive assessment. Current IQ was estimated from the Wechsler Adult Intelligence Scale (WASI); subscale similarities and block design. Verbal learning was measured with the California Verbal Learning Test (CVLT-II) [32], verbal learning sub-score. Processing speed was measured with the Digit Symbol Test [33]. Working memory was measured with the Letter number sequencing test [33]. Verbal fluency was measured with the Verbal Fluency Test, phonetic sub-score from the Delis–Kaplan Executive Functioning System (D-KEFS) [34]. Finally, inhibition was measured with the Color-Word Interference Test from the D-KEFS [34], inhibition sub-score. Raw scores were reported for all tests to be able to calibrate the cognitive performance of the FEP patients compared to a representative age- and gender-matched HC group from the same catchment area as the patients. Higher scores indicated better performance on all tests except for the inhibition sub-test where higher scores indicated poorer performance. All participants showed an adequate neuropsychological test effort indicated by two errors or less on the forced recognition trial of the California Verbal Learning Task (CVLT-II) [32].

### Serum lipids and BMI

Blood was drawn from the antecubital vein in the morning after overnight fasting at baseline and 12 months, on the same day as the neuropsychological testing took place (time period: 2003–2013). The Department of Clinical Chemistry at Oslo University Hospital, using standard enzymatic methods from Roche Diagnostics Norge AS (Oslo, Norway), analyzed cholesterol (total, HDL, LDL) and triglycerides (TG). All blood samples were sent immediately after collection and analyzed continuously as routine samples. Digital weights were used to weight all the participants and body mass index (BMI) calculated accordingly: BMI = weight (in kilograms) divided by height (in meters squared) (kg/m^2^).

### Statistical analyses

The data were analyzed with SPSS version 23 (SPSS Inc., Chicago, IL, USA/IBM, New York, USA). The level of statistical significance was preset to *p* < 0.05 (two tailed). For the comparison of demographic characteristics, chi-square tests and *t* tests were used as appropriate.

For the main analyses, we employed linear mixed models with unstructured covariance matrix to examine longitudinal changes and between-group effects of lipid measures on cognitive measures. Preliminary analyses were conducted to ensure assumptions of normality, linearity, multicollinearity, and homoscedasticity. Serum TG values were ln-transformed after inspection of residuals. Due to issues with multicollinearity, we did not enter all the lipids in the same mixed model analysis. There were no problems with linearity or homoscedasticity.

In separate mixed models, we first examined any baseline differences in metabolic and cognitive measures controlling for age, gender, and education. Then, we explored the 1-year changes in serum lipids, BMI, and cognitive performance. In the latter longitudinal models, we also included time and a group (FEP and HC) × time interaction.

Our approach to the interpretation of statistical results obtained from the cognitive models was that a main effect of time would indicate cognitive changes due to drug treatment or other causes (i.e., practice effects). To disambiguate the latter two possibilities, we would compare the cognitive performance of the patient group with that of the HC group. Group × time interactions that favored steeper improvement in the FEP group would be viewed as an indication of a possible treatment effect, reflecting cognitive enhancement. A main effect for time in the absence of such an interaction could be regarded as representing practice effects.

The mixed models that displayed a significant group × time interaction were subsequently examined for a potential relationship with serum lipids. The rationale for this was that group-specific changes to cognition might be related to alterations in serum lipids due to antipsychotic drug treatment. In these mixed models, the scores from the specific cognitive tests were defined as dependent variables, while time (baseline and 12 months), age, gender, years of education, group affiliation (FEP or HC), and serum lipids were entered as independent variables. To investigate if there was a group-dependent effect of lipids on cognition, we also included a group × lipid interaction.

In post hoc analyses, using similar mixed models as in our main analyses, we further examined if any group-specific lipid effects on cognition could be explained by ethnicity, lifestyle-related factors (smoking, alcohol, diet, and exercise), concomitant changes in BMI, be secondary to antipsychotic treatment effects on positive and negative symptoms, remission status at 12 months, or be explained by other illness-related factors (hospitalization and depressive symptoms).

### Missing data

Preliminary analyses indicated a random distribution of the missing variables and that there were no significant differences in sociodemographic characteristics or cognitive test scores between the subjects with a complete dataset on serum lipids compared to the ones with missing data (Little’s MCAR test: chi-square = 1912.51, *df* = 1865, *p* = 0.22). Forty-seven (36%) of the FEP patients lacked cognitive data at follow-up. These FEP patients were included in the main analyses because they had baseline data and a mixed linear model approach using all available information was employed. As a precaution, we also checked the validity of our results by repeating the analyses excluding participants with an incomplete dataset.

## Results

For a detailed description of demographic characteristics and group comparison of FEP with HC, see Table 1. In summary, the FEP patients had significantly shorter education, more non-Caucasian ethnicity and a higher percentage of daily smokers, compared to the HC.

### Group differences in serum lipids at baseline and after 1 year of follow-up

As described in Table 2, the FEP patients had significantly higher levels of total cholesterol and LDL compared to the HC at baseline, (*F *= 8.94, *p *= 0.003) and (*F *= 11.54, *p *= 0.001), respectively. During the follow-up, there were no significant changes in mean levels of serum lipids among the FEP patients when compared to the HC at the group level (Table 3). Still, there were individual differences, and at the individual level the serum lipid changes for the FEP were the following: 47 participants (47%) experienced a decrease and 52 participants (53%) an increase in their total cholesterol levels; 49 participants (54%) experienced a decrease and 41 participants (46%) an increase in their HDL levels; 43 participants (50%) experienced a decrease and 43 participants (50%) an increase in their LDL levels; 40 participants (45%) experienced a decrease and 49 participants (55%) an increase in their TG levels.

### Group differences in cognition at baseline and after 1 year of follow-up

The FEP patients demonstrated marked impairments in cognitive functioning across all domains at baseline (*p *< 0.001) (Table 2). During the 1-year follow-up, there was a main effect of time (*F *= 17.60, *p *< 0.001) (indicating significant changes in cognitive performance from baseline to 12 months) as well as a group by time interaction for verbal learning (*F *= 6.86, *p *= 0.01), with a steeper change in FEP compared to HC (*B *= 3.54, *p *= 0.01), indicating that practice effects alone cannot explain the relatively higher improvement in patients compared to HC. The mixed model analyses also showed a group by time interaction for verbal fluency (*F *= 6.38, *p *= 0.01); here, the FEP group exhibited a deterioration in comparison with the HC group, (*B *= − 3.15, *p *= 0.01), respectively.

### Association between serum lipids and cognition

When examining the two cognitive domains that showed a group by time interaction (verbal learning and verbal fluency) for associations with serum lipid levels, we found that there was a significant group by HDL interaction effect for verbal learning (*F *= 11.12, *p *= 0.001), where an increase in HDL level was related to improvement in verbal learning among FEP patients compared to the HC (*B *= 10.32, *p *= 0.001). Verbal fluency, in contrast, did not evidence any significant group by lipid interactions. Follow-up analyses excluding patients with incomplete cognitive datasets produced comparable results (data not shown).

Because the FEP patients, in contrast to HC, included several subjects with their primary education outside Norway, which could influence the cognitive test scores, we also reran the analyses without these patients and obtained similar results as in our main analyses (data not shown).

### Post hoc analyses exploring the significant associations found between serum lipids and cognition

The post hoc mixed models controlling for antipsychotic usage (i.e., continuous use vs. intermittent use), and positive and negative symptoms indicated that the group (i.e., FEP)-specific link between HDL and verbal learning remained highly significant (*F *= 7.45, *p *= 0.007). A significant main effect of positive symptoms (*F *= 4.67, *p *= 0.03) and negative symptoms (*F *= 11.77, *p *= 0.001), but not for antipsychotic drug use (*F *= 1.03, *p *= 0.31), was also exhibited. As the magnitude of the effect is reflected in the standardized regression coefficients, we examined these coefficients to discern whether HDL cholesterol had a stronger impact than negative and positive symptoms on verbal learning. Our results indicated that HDL (*B *= 3.13, *p *= 0.007) was a stronger predictor for verbal learning than were negative (*B *= − 2.20, *p *= 0.001) and positive symptoms (*B *= − 1.76, *p *= 0.03). Likewise, controlling for remission status did not significantly impact the observed link between serum HDL and verbal learning in the FEP group (*B *= 8.42, *p *= 0.002).

We further investigated the influence of antipsychotic drug treatment (continuous use vs. intermittent use, and antipsychotic monotherapy vs. polypharmacy) on the relationship between HDL levels and verbal learning. The group (FEP)-specific HDL-link remained highly significant, (*F *= 10.76, *p *= 0.001) and (*F *= 10.54, *p *= 0.001), whereas the main effect of antipsychotic drug treatment was absent, inferring limited value to differential treatment effects on the observed link between HDL and verbal learning.

Furthermore, though there was a main effect of BMI on verbal learning (*F *= 4.88, *p *= 0.03), the group by HDL interaction remained significant (*F *= 9.86, *p *= 0.002) indicating that the link between HDL and verbal learning was independent of BMI. Of note, ethnicity, hospitalization, depressive symptoms, smoking habits, diet, exercise, and alcohol use demonstrated no attenuation of results for the observed link between HDL and verbal learning (data not shown).

## Discussion

The current study of antipsychotic-treated FEP patients showed that during the first year of follow-up, an increase in serum HDL was associated with better verbal learning capacity. This relationship was independent of practice effect (as controlled by a group of age- and gender-matched HC), lifestyle, concurrent changes in BMI, and improvements in clinical symptomatology. Having in mind that cognitive performance is an important predictor of longitudinal clinical and functional outcomes in patients with psychotic disorders, our findings may be of clinical relevance.

Consistent with prior literature [35], the FEP patients performed poorer on all cognitive tests compared to the HC. During the follow-up, there was a group-specific improvement in verbal learning, which subsequent analyses showed was associated with an increase in HDL levels among the FEP patients but not amongst HC. Our findings are in line with Lancon and colleagues [18] finding a positive association between HDL and verbal learning and memory in a cross-sectional sample of chronic schizophrenia patients. The present study is, however, the first to report such an association in a cohort of FEP patients with age- and gender-matched healthy controls.

The observed link between HDL and cognition is also consistent with the mechanisms that relate lipids to the brain while also agreeing with evidence of impaired lipid metabolism (including cholesterol) in psychotic patients [36–40]. Lipids are not only essential for myelination; synaptic growth and regeneration also depend greatly on the availability of brain cholesterol [41, 42]. Depletion of cholesterol in tissue cultures has, for example, been shown to inhibit synaptogenesis and causes neurodegenerative changes [42]. Brain structures such as the hippocampus express high transcript levels of lipid-biosynthesizing enzymes [42], are densely populated by synapses, and play a prominent role in verbal learning and memory [43, 44]. Several studies have found a positive link between performances on verbal learning and hippocampal volumes [45–47], as well as on distinct subfield volumes [48]. More importantly, a growing number of evidence in the normal aging population and patients with neuropsychiatric disorders, points to high serum HDL levels, in particular, being protective against hippocampal atrophy [49]. This dependency of the hippocampus on intact cholesterol metabolism may at least in parts explain our finding of a distinct link between HDL cholesterol and the hippocampus-based task of verbal learning. While studies are reporting a correlation between different cognitive domains within an individual [50–55], there are several investigators that have emphasized a cognitive architecture best described by relatively independent cognitive domains [2, 56–58], and verbal learning is recognized as one such independent cognitive dimension. In further support of distinguishable cognitive dimensions is the fact that pharmacological interventions, including antipsychotic drug treatment, have been shown to cause changes in specific cognitive domains [59], indicating a pharmacological dissociation of certain groups of cognitive processes.

For decades, the traditional view has been that the cholesterol in the brain is synthesized de novo; newer evidence, however, suggests that excessive cholesterol synthesis may be regulated by intricate efflux mechanisms linking central and peripheral cholesterol levels [60–63]. Indeed, serum HDL has been found to be highly correlated with HDL in cerebrospinal fluid [64]. Still, the nature of this relationship, between serum lipids and lipids in the CNS, is not fully elucidated. It is, however, likely that long-term lipid status may have a different impact on brain structure and functioning than the short-term impacts observed in treatment studies [19, 20, 65], and that these changes in brain structure and functioning may be more pronounced in the developmental lifespan [66]. While we included a relatively young FEP population (with a mean age of 25 years), future studies may consider including high-risk patients, as well as having a longer study period with frequent observation points.

We recently demonstrated that an increase in HDL during antipsychotic drug treatment was related to an improvement in negative symptoms in the same FEP cohort [25]. Other studies have shown a relationship between negative symptoms and verbal learning [67]. As we now find HDL levels being associated with specific cognitive domains, it is plausible that improvements in cognitive functioning over time were more related to clinical symptom improvement, reflecting “pseudospecificity” [68]. Additionally, lack of motivation and motor retardation inherent in the negative syndrome could also have disturbed test performance in patients with prominent negative symptoms. We attempted to disentangle these phenomena by controlling for secondary changes in negative (and positive) symptoms during antipsychotic drug treatment, obtaining similar results as in our main analyses. Intriguingly, the standardized regression coefficients revealed that the HDL level was more profoundly related to working memory than positive and negative symptoms were, adding support to the growing literature on the role of lipids in psychotic disorders. Also, other factors that are known to influence cholesterol metabolism as well as cognitive function such as lifestyle and behavioral factors [69–73] were controlled for in the post hoc analyses, achieving similar results as in our main analyses.

The present study has some additional limitations that need to be addressed. The constitution of the HC group was different from the FEP group since the latter included subjects with their primary education from outside Norway, which could influence cognitive performance especially for verbal tests. We tried to minimize the effect of this limitation by controlling for education in the mixed model analyses. Also, we reran the analyses excluding the patients without a Norwegian education, generating similar results. Information on diet and exercise habits was only available for FEP but not for HC. Additionally, self-assessment about diet and exercise may be problematic as it may be linked to high social desirability; on the other hand, achieving truly reliable data on these parameters may be difficult outside the confinement of an institution. Due to power issues, we were not able to examine specifically and compare different antipsychotic agents and how this may influence our results. Still, our main objective was to examine the effect of serum lipids per se on cognition in FEP patients, and for this particular aim, differentiating the antipsychotic agents might be less prudent. We further recognize that other factors then practice can explain a main effect of time: the controls are healthy, and could in theory be susceptible to a ceiling effect in the amount of change that can be achieved; leading to potential differences in the learning potential in the two groups. While an untreated control group may have been ideal to address this matter, it is not ethically feasible. Finally, as our study is naturalistic in design, temporality cannot be established, and studies with a randomized controlled design, which include treatment targeted explicitly at elevating HDL levels and measurements of change in cognitive performance, are necessary.

In conclusion, the present study of antipsychotic-treated FEP patients showed that during the first year of follow-up, an increase in serum HDL was associated with better cognitive performance. Further research, implementing clinical data with for instance myelin-focused brain imaging techniques, is warranted. As cognitive deficits dramatically impact social and occupational functioning, our findings of a link between HDL and cognition could potentially contribute to the development of new treatment strategies, with functional improvements achieved by increasing HDL levels as an add-on treatment for cognitive remediation that has shown to be effective in patients with psychosis.

## Electronic supplementary material

Below is the link to the electronic supplementary material.

Supplementary material 1 (DOCX 292 kb)
