# Supplementary material for: Improvement in verbal learning over the first year of antipsychotic treatment is associated with serum HDL levels in a cohort of first episode psychosis patients
Source: Eur Arch Psychiatry Clin Neurosci. 2019 Apr 27;270(1):49–58. doi: 10.1007/s00406-019-01017-w (PMC7033047; doi:10.1007/s00406-019-01017-w)
Supplement: Supplementary file 1 [file 406_2019_1017_MOESM1_ESM.docx]

Supplementary Table 1: Antipsychotic drug use of each patient at baseline and at 12 months

Antipsychotics at baseline			Antipsychotics at 12 months	
1. antipsychotic agent	2. antipsychotic agent	3. antipsychotic agent	1. antipsychotic agent	2. antipsychotic agent
Amisulpride			Amisulpride	
Aripiprazole			Aripiprazole	
Aripiprazole			Quetiapine	Clorprothixene
Aripiprazole	Olanzapine		Aripiprazole	Quetiapine
Aripiprazole	Risperidone inj.		Quetiapine	
Aripiprazole			Olanzapine	
Aripiprazole	Olanzapine		Olanzapine	Zuclopenthixol
Aripiprazole				
Aripiprazole			Aripiprazole	
Aripiprazole			Olanzapine	
Aripiprazole	Olanzapine		Quetiapine	Risperidone inj.
Aripiprazole	Clorprothixene			
Aripiprazole			Aripiprazole	
Aripiprazole			Aripiprazole	
Aripiprazole	Clorprothixene		Aripiprazole	
Aripiprazole			Aripiprazole	
Aripiprazole	Olanzapine		Olanzapine	
Aripiprazole			Sertindole	
Aripiprazole	Olanzapine		Aripiprazole	
Aripiprazole				
Aripiprazole				
Aripiprazole				
Aripiprazole	Olanzapine	Zuclopenthixol	Sertindole	
Aripiprazole			Aripiprazole	
Aripiprazole				
Aripiprazole			Aripiprazole	
Flupentixol			Zuclopenthixol	
Olanzapine			Risperidone	
Olanzapine			Olanzapine	
Olanzapine	Aripiprazole		Clozapine	Olanzapine
Olanzapine			Olanzapine	
Olanzapine			Quetiapine	
Olanzapine			Aripiprazole	
Olanzapine			Aripiprazole	
Olanzapine			Aripiprazole	
Olanzapine			Aripiprazole	
Olanzapine			Olanzapine	
Olanzapine			Quetiapine	Risperidone inj.
Olanzapine			Risperidone inj.	
Olanzapine			Olanzapine	Risperidone
Olanzapine			Olanzapine	
Olanzapine	Risperidone		Risperidone inj.	
Olanzapine			Olanzapine	
Olanzapine			Perhenazine	
Olanzapine			Aripiprazole	
Olanzapine			Olanzapine	
Olanzapine	Clorprothixene		Olanzapine	Clorprothixene
Olanzapine			Perhenazine deconate	
Olanzapine			Ziprasidone	
Olanzapine			Olanzapine	
Olanzapine			Ziprasidone	Clorprothixene
Olanzapine			Olanzapine	
Olanzapine			Olanzapine	
Olanzapine			Olanzapine	
Olanzapine			Olanzapine	
Olanzapine			Aripiprazole	
Olanzapine				
Olanzapine			Olanzapine	
Olanzapine			Aripiprazole	
Olanzapine			Quetiapine	
Olanzapine				
Olanzapine			Aripiprazole	Olanzapine
Olanzapine	Chlorpromazine	Levomepromazine		
Olanzapine			Olanzapine	
Olanzapine	Risperidone		Aripiprazole	
Olanzapine	Levomepromazine		Quetiapine	
Olanzapine				
Olanzapine			Olanzapine	
Olanzapine			Aripiprazole	
Olanzapine	Risperidone		Quetiapine	Aripiprazole
Olanzapine			Aripiprazole	
Olanzapine	Paliperidone		Aripiprazole	
Olanzapine			Aripiprazole	Olanzapine
Paliperidone			Paliperidone	
Perhenazine			Aripiprazole	
Perhenazine			Quetiapine	
Perhenazine decanoat			Olanzapine	
Quetiapine	Aripiprazole			
Quetiapine	Aripiprazole		Aripiprazole	Quetiapine
Quetiapine				
Quetiapine			Aripiprazole	Risperidone inj.
Quetiapine	Olanzapine	Clorprothixene	Risperidone	
Quetiapine	Amisulpride	Olanzapine	Quetiapine	Amisulpride
Quetiapine			Quetiapine	
Quetiapine				
Quetiapine	Aripiprazole			
Quetiapine	Olanzapine		Quetiapine	
Quetiapine	Olanzapine		Quetiapine	
Quetiapine			Ziprasidone	
Quetiapine			Quetiapine	Aripiprazole
Quetiapine			Quetiapine	
Quetiapine				
Quetiapine			Quetiapine	
Quetiapine	Risperidone	Clorprothixene		
Quetiapine				
Quetiapine			Quetiapine	
Quetiapine	Olanzapine		Clozapine	Aripiprazole
Risperidone			Quetiapine	
Risperidone	Risperidone		Risperidone inj	
Risperidone			Risperidone	
Risperidone				
Risperidone			Aripiprazole	
Risperidone			Aripiprazole	
Risperidone			Olanzapine	
Risperidone	Levomepromazine		Risperidone	Olanzapine
Risperidone			Aripiprazole	
Risperidone			Clozapine	
Risperidone				
Risperidone				
Risperidone inj.			Risperidone	
Risperidone inj.			Risperidone inj.	
Ziprasidone			Ziprasidone	
Ziprasidone			Ziprasidone	
Ziprasidone	Zuclopenthixol		Aripiprazole	Quetiapine
Ziprasidone			Aripiprazole	
Ziprasidone			Ziprasidone	
Ziprasidone	Chlorpromazine		Quetiapine	Ziprasidone
Ziprasidone			Olanzapine	
Ziprasidone	Olanzapine		Quetiapine	
Ziprasidone			Aripiprazole	Olanzapine
			Olanzapine	
			Aripiprazole	
			Amisulpride	Ziprasidone
			Risperidone inj.	
			Olanzapine	
			Clorprothixene	
			Perhenazine	
			Aripiprazole	
			Aripiprazole	
			Quetiapine	
			Aripiprazole	Quetiapine
			Aripiprazole	

This table shows the antipsychotic drug(s) used by each FEP patient (i.e. the combination of drugs used by each individual).

Supplementary Table 2: Antipsychotic use in the FEP sample

	Antipsychotic agent at baseline									Antipsychotic agent at 12 months			
	1. antipsychotic agent			2. antipsychotic agent			3. antipsychotic agent			1. antipsychotic agent		2. antipsychotic agent	
	n	Mean dose (SD)	Mean duration (SD)	n	Mean dose (SD)	Mean duration (SD)	n	Mean dose (SD)	Mean duration (SD)	n	Mean dose (SD)	n	Mean dose (SD)
**Typical antipschotics ***													
Flupentixol	1	40	missing b, #										
Zuclopenthixol				1	9.50	2	1	37.10	0			1	10
Zuclopenthixol deconate										1	200.00		
Chlorpromazine				2	8.75 (1.77)	3							
Perphenazine	2	12.00 (5.66)	6.4 (7.6)							2	12.00 (5.66)		
Perphenazine deconate	1	5.15	1.5							1	7.70		
Levomepromazine				2	55.00 (63.64)	0.2	1	25.00	5				
Chlorprothixene				3	26.67 (20.21)	0.6 (0.4)	2	57.50 (60.10)	0.6 (0.6)	1	25.00	3	2.33 (184.28)
**Atypical antipsychotics ***													
Olanzapine	46	12.99 (5.76)	3.5 (5.0)	11	10.45 (7.31)	2.6 (2.1)	1	12.5	3	25	14.4 (7.68)	5	7.50 (3.95)
Quetiapine	20	450.00 (299.23)	2.1 (3.2)							21	494.05 (396.38)	4	175 (95.74)
Risperidone	12	3.06 (1.56)	2.3 (2.3)	5	4.40 (1.67)	0.4 (0.2)				5	3.50 (1.91)	1	3
Risperiodone injection	2	2.25 (0.64)	0.9 (1.0)	1	missing c, #	5				5	3.60 (0.00)	3	3.30 (0.52)
Aripiprazole	25	14.17 (3.81)	2.3 (3.1)	4	8.75 (4.79)	3.5 (1.0)				37	14.33 (6.97)	3	30.00 (13.23)
Ziprasidon	9	77.78 (48.42)	5.4 (5.2)							6	56.67 (42.74)	2	90 (98.99)
Paliperidone				1	3.30	0.5							
Paliperidone injection	1	missing a, #	missing a, #							1	100		
Sertindole										2	12.00 (11.31)		
Amisulpride	1	400	2	1	500	0.3				2	400 (0.00)	1	500
Clozapine										3 d	233.33 (152.75)		

Mean duration is reported in weeks. Mean dose is reported in mg. n= number of patients. SD= standard deviation.
We did not have data on duration of antipsychotic treatment at 12 months.
Typical antipsychotic agents were always used with atypical antipsychotic agents.
* Monotherapy at baseline and/or 12 months (but not necesserily with the same antipsychotic agent): n=90. Of these, monotherapy with the same antipsychotic agent at baseline and at 12 months (n): olanzapine (15), aripiprazole (7), quetiapine (4), risperidone (tablet or injection) (3), ziprasidone (3), and paliperidone (1). Polypharmacy: n= 42
a) paliperidone (xeplion): although missing dosage and duration, the patient used this depot injection continously, and at 12 months used 100 mg which was also confirmed by levels of antipsychotics in serum.
b) this patient used flupentixol at baseline, which was confirmed by detectable levels of antipsychotics in serum, then switched to zuclopenthixol injection during the follow-up period.
c) missing data (25 mg i.m is the standard dose).
d) Among the clozapine users, one was on monotherapy, while two patients used clozapine in combination with olanzapine or aripiprazole.
reanalyzing while excluding these patients did not change the results significantly.

Supplementary Table 3: Co-medication at baseline in the FEP sample

Active substance	Baseline (n)
**Antiepliecticum**	
Lamotrigine	4
Gabapentin	1
Clonazepam	5
Carbamazepine	1
**Antidepressants**	
Escitalopram	17
Citalopram	4
Venlafaxine	8
Fluoxetine	3
Mirtazapine	4
Amitriptyline	1
Paroxetine	2
Sertraline	4
Duloxetin	1
**Anxiolytics, sedatives and hypnotics**	
Zopiclone	5
Oksazepam	2
Alimemazine	2
Diazepam	1
**ADHD**	
Methylphenidate	1
**Other regular medications**	
Alendronate	1
Antihistamines	3
Atenolol	1
Folic acid	1
Ibuprofen	1
Insulin human (rDNA)	1
Levothyroxine	1
Medroksyprogesteronacetat	1
Mometasone furoate	1
Oxycontin	1
Pantoprazole	1
Tetracycline	1
Warfarin	1

This table shows both the psychotropic drugs and the non-psychotropic drugs used at baseline by the FEP patients. n= number of patients.
